# HBx M130K and V131I (T-A) mutations in HBV genotype F during a follow-up study in chronic carriers

**DOI:** 10.1186/1743-422X-2-60

**Published:** 2005-08-04

**Authors:** Bernal León, Lizeth Taylor, Minor Vargas, Ronald B Luftig, Federico Albertazzi, Libia Herrero, Kirsten Visona

**Affiliations:** 1 International Center for Medical Research and Training, Louisiana State University ICMRT-LSU, San José, Costa Rica; 2 Pathology Department, San Juan de Dios Hospital, CCSS, Costa Rica; 3 Molecular Biology Center, Universidad of Costa Rica; 4 Virology Department, Microbiology School, Universidad of Costa Rica; 5 Microbiology, Immunology & Parasitology Department, School of Medicine, Louisiana State University, USA

## Abstract

**Background:**

Around 400 million people worldwide are chronically infected with Hepatitis B virus (HBV). An estimated 10% of these chronic patients develop progressive liver damage including cirrhosis and Hepatocellular Carcinoma (HCC). The HBx gene encodes a protein of 154 amino acids which is a transactivator and has been associated with HBV pathogenesis. A change in the amino acid sequences at positions 130 and 131 in the HBV-X protein (M130K and V131I) produced by T-A point mutations at the nucleic acids level has been associated with severe liver damage and HCC in patients from China and Africa. Further, such changes have been proposed as a prognostic marker for progressive liver damage and HCC. The purpose of this study was to determine if T-A mutations are present in HBV chronic carriers with genotype F (the major genotype in Costa Rica) and further, if these mutations are associated with HBV disease progression in Costa Rica HBV patients from 1972 to 1985.

**Results:**

Serum samples from 50 HBV positive individuals were amplified and directly sequenced, 48 belonged to genotype F, 1 from genotype D and another was classified as D or E.

T-;A mutations were absent in 17 acute patients who recovered, but was present in 12 of 29 chronic carrier samples (42.8%), in one sample the T-A mutations were detected as early as 29 days after clinical onset of disease. In 17 carriers with available liver biopsies, T-;A mutations were found in 8 sera of 13 (61.5%) classified as moderate or severe, and none in 4 biopsies with mild liver damage. However, it was not possible to demonstrate a statistical association between the presence of T-A mutations and moderate/severe liver damage, using a Fischer exact test, 1 tail, p = 0.05.

In 4 patients HCC was diagnosed, and 2 of them presented the T-A mutations in their sera.

**Conclusion:**

T-A mutations were found in HBV genotype F in chronic carriers but not in patients who recovered from acute infection. These mutations could be developing early during infection although the possibility of infection with the mutant virus could not be excluded.

More studies are necessary to establish if the T-A mutation can be used as a prognostic marker for severity of liver disease in patients infected with HBV.

## Background

The hepatitis B virus (HBV) is a small double stranded DNA virus that produces a chronic infection in 2–10% of adults and in approximately 90% of infected infants. Approximately 10% of these chronic patients develop progressive liver damage including cirrhosis and Hepatocellular Carcinoma (HCC)[1]. The mechanism by which HBV progression to liver cirrhosis and/or HCC occurs is not clear, however many studies suggest that the X protein (HBx) is related to this process. HBx has been associated with a variety of biological functions. As a transcriptional transactivator, it can regulate transcription of a wide diversity of viral and cellular promoters [2,3]. HBx overlaps with regions of crucial importance for viral replication such as: the direct repeat sequences DR1 and DR2, the preC/C gene promoter and the enhancer II region. There are controversial results about the consequence of mutations in this region and its relationship with pathogenesis. A study carried out in Korea determined that mutations in the core promoter have little effect on viral load and the HBeAg status [4]. In contrast, another study points out that changes in HBx especially in the core promoter region may alter HBV gene expression [5]. Among other alterations observed in the HBx gene are deletions and one of the most common is the 8 bp deletion between nucleotides 1763–1770 [6], which has been described to decrease the virus replication [7,8]. These deletions in HBx as in other HBV genes have also been related to development of cirrhosis in long term renal transplant patients [9].

Natural mutations in the HBx gene have been related to progression to chronic disease as a consequence of the rescission of anti proliferative and apoptotic effects, which might produce uncontrolled growth and contribute to multistep hepatocarcinogenesis [10].

A double point mutation with a transversion nucleotide from adenine to thymine at nucleotide 1762, K130M with a transition from adenine to guanine at position 1764 V131I (T-A mutations), has been found more frequently in patients with hepatic tumors than in asymptomatic chronic patients from China [11,12] and Africa [13]. In East Asia where genotype C is the most common genotype, it has been reported that the T-A mutation occurs more frequently in relation to this genotype [14]. HBV is classified worldwide into eight genotypes designated A to H, with a specific geographical distribution [15-17].

Genotype F has been described as the HBV genotype of the Amerindians. In Central America a study determined 79% of samples belong to genotype F [18] and in Costa Rica genotype F is the most common, while the overall prevalence of HBV is considered low (0.5 – 1%).

From 1972 to 1985 a study on the natural history of HBV was done in San Ramón and Palmares, two adjacent Costa Rican counties [19]. In this study 488 cases of HBV were diagnosed, 80% with an age range between 5 and 40 years. In the group ≤ 5 years old 33% became chronic carriers and in the group > 5 years only 4.7% did. The 77.7% cases were primary HBV infections and the rest were due to household contacts. The purpose of this study was to analyze the presence of T-A mutations in the HBx gene for this population; the time which at they occur and if they are related to hepatic injure. Furthermore, the presence of other mutations in this gene were also observed

## Results

### PCR detection rate

Of the 77 selected samples, 18 were from group A, 14 from group B and 45 from group C; overall, 50 samples (64.9%) could be amplified and sequenced. Of these fifty, 17 (94.4%) were from group A (recovered patients), 12 (85.7%) from group B (paired samples – known onset), and 21(46.6%) from group C (chronic patient with unknown onset). The sensitivity of the nested PCR was 8000 copies/ml.

### T-A mutations were present in chronic HBV carriers but not in acute recovered patients

Table 1 shows the mutation rate of T-A in HBx for M130K and V131I amongst the three study groups. The T-A mutations were not present in any of the 17 sequences from group A, where the average days in which samples were taken was 17 days ranging from 3–33 days. Of 8 chronic patients in group B, the T-A mutations were identified in 5 (62.5%) of the sequenced samples and V131I alone was detected in two. In one of the patients, T-A mutations were detected at day 29 after clinical onset. Four patients were not considered in the distribution of T-A mutations, since the follow-up samples could not be amplified. From group C the T-A mutations were detected in 7 of the 21 sequenced samples, and V131I alone in 3 samples.

**Table 1:** Distribution of the T-A mutations leading to (K130M and V131I) in the study groups.

**GROUP**	**MUTATIONS**			
	**T-A mutations**		**V131I alone**	
	**#/n**	**(%)**	**#/n**	**(%)**
A	0/17	-	-	
B	5/8	(62.5)	2*/8	
C**	7/20	(31.8)	3/20	

* One of these two sample had V131I mutation in the first sample and TGA mutations later after a five year interval. One sample presented a deletion in that position

** One sample presented a deletion in the T-A position

### Biopsy results and T-A mutations distribution

**Figure 1:**
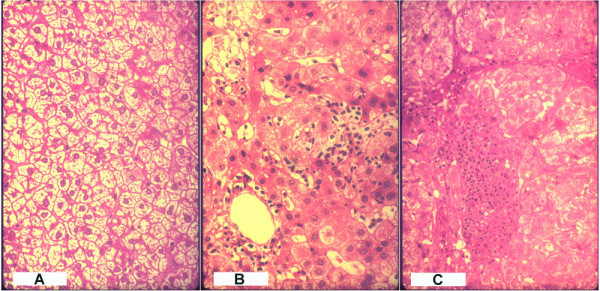
**(A) – Persistent chronic hepatitis, Knodell index ≤ 2**. Photomicrograph of liver showing chronic hepatitis with minimal activity. Hepatocytes showing regenerative features are seen, with minimal inflammation and scattered ground- glass hepatocytes. Cobblestone arrangement (diffuse regeneration) with Hadziyannis cells and without necrosis or fibrosis. (H&E 250×). **(B) – Mild lobular chronic hepatitis, Knodell index 3–4**. Photomicrograph of liver showing chronic hepatitis with mild activity. Spotty hepatocyte necrosis is seen in a lobular pattern with focal lymphocytic infiltration. Lesions are characterized by focal necrosis, conserved sinusoidal and trabecular patterns, lobular, portal, and focal lymphocytic infiltrated. (H&E 400×). **(C) – Moderate lobular chronic hepatitis, Knodell index > 4**. Photomicrograph of liver showing chronic hepatitis with moderate activity. There is portal chronic inflammation, focal interface hepatitis and periportal fibrous septa. Portal chronic swollen periportal apoptosis, post-necrosis fibrous interportal bridges. Nodular regeneration (pre-cirrhosis). (H&E 250×)

Of the 29 chronic carrier samples from groups B and C sequenced during the chronic phase, 18 patients had a liver biopsy characterized using the Knodell Index (KI). Five (26%) patients had a KI ≤ 2 points, (mild liver lesions with fatty deposits), 9 (47%) had KI between 3 and 4 points (moderate lesions) and 4 (21%) had a KI > 4 points (severe lesions). These are shown in fig. 1a, 1b and 1c respectively.

**Figure 2:**
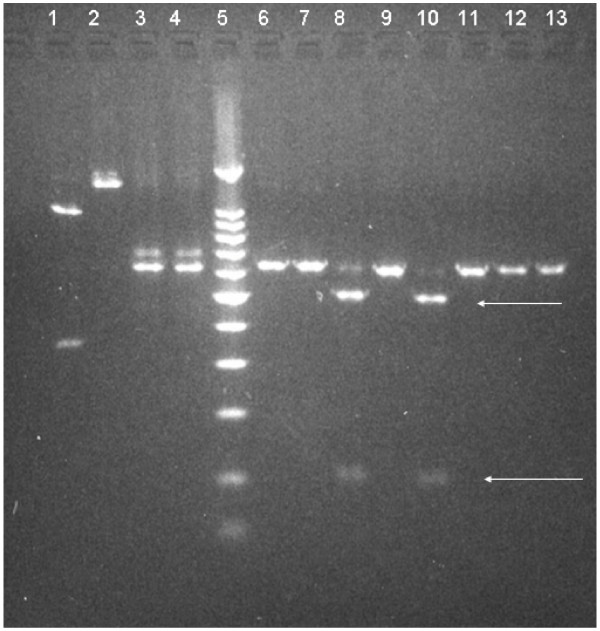
**Sample deletions treated with Ssp I restriction enzymes**. Recognition site of the enzyme SspI in the sequences with 8 bp deletion (left). In the right, samples with presumed deletions were run in a 3% agarose gel. Each pair of lines have the same sample treated with and without the Ssp I enzyme. An HIV sample having the AATATT site was used as positive control in lanes 1 and 2, sample 1430 (616 bp) lanes 3 and 4 (negative control), 1000 bp ladder marker lane 5, sample 6290 lanes 6 and 7, sample 467 lanes 8 and 9 sample 6516 lines 10 and 11, sample 6541 lanes 12 and 13. The samples 467 and 6516 treated with SspI presented two bands of 507 and 109 bp, lanes 8 and 10 (arrows) confirming the deletion. Details in sequence are:

Of the 5 carriers with biopsy classified as KI ≤ 2, one sample had an 8 bp deletion that included the T-A mutations site and another sample the V131I mutation alone. In the group with KI > 2 points (moderate/severe) T-A mutations were present in 8 (61.5%) of the sequenced samples (Table 2).

**Table 2:** Correlation between Knodell Index (KI) and HBx-T-A mutations.

	**MUTATIONS**			
	**T-A mutations**		**V131I alone**	
**Results KI**	**#/n**	**(%)**	**#/n**	**(%)**
≤ 2	0/4	-	1/4	-
> 2	8/13 *	(61.5)	-	

* One sample presented a deletion in the T-A position Fisher exact test, 1 tail, M130K p = 0.05, V131I p = 0.24

Table 3 reveals HBV carrier biopsies with KI > 2, age of the carrier at time of biopsy and sample collection, TSGO/TSGP levels and HBeAg/anti-HBe status.

According with statistics of the Costa Rican National Tumor Registry (NTR), four patients included in this study died from HCC during the last 2 decades and two of these had the presence of T-A mutations.

**Table 3:** Characterisation of samples with biopsies considered moderate and severe and patients who died from HCC.

Sex/Group	Patient ident	Sample Id/Time of sample collection after onset or study initiation	Age at time of sample collection	Patient age at time of biopsy collection	Knodell Index	K130M/V131I	HBeAg/Anti-HBe	TSGO/TSGP
M/B	950-08	865M/2 y	4			-/-	+/-	50/32
		6217M/12 y	14	15	2+1 = 3	+/+	-/-	
								
M/B	671-10	445E/19 d	8			-/-	+/-	74/83
		5252M/9Y	17	18	2+2 = 4	+/+	-/+	32/18
								
M/C	266-03	3751M/8 m	35			-/-	-/+	32/13
		6400M/6 y	40	40	3+3 = 6	+/+	-/-	36/13
								
M/B	496-04	6461E/2 y	12			-/+	-/-	45/40
		4904M/7 y	17	19	2+1 = 3	+/+	-/+	28/25
								
M/B	969-04	467H/23 d	24			deletion	-/-	500/550
		6604M/7 y	31	32	HCC 5+2 = 7	+/+	-/-	ND/18
								
M/C	1232-06	1481M/10 m	12			-/+	-/+	36/21
		6290M/9 y	22	23	2+1= 3	-/-	-/+	
								
M/C	65-35	6891M/11 y				+/+	-/+	55/50
			20	20	3+0 = 3			
								
M/C	158-01	6151M/11 y				+/+	-/+	36/16
			33	35	1+2 = 3			
								
M/C	921-07	6403M/10 y				+/+	-/-	28/21
			31	32	3+1 = 4			
								
M/C	673-04	6572M/8 y				-/-	-/-	ND/9
			25	26	3+1 = 4			
								
M/C	5-02	6067M/9 y			2+2 = 4	-/-	-/+	32/16
			53	54				
								
M/C	671-06	6593M/15 y				-/-	-/-	ND/13
			26	26	4+3 = 7			
								
M/C	671-09	5251M/9y			2+4 = 6	-/-	-/+	28/18
			16	19				
								
M/B	1688-16	3254H/3 d	3		HCC	-/-	-/-	475/225
	16	6653M/3 y	6		NB	+/+	-/-	ND/36
								
M/B	1400-01	575H/2 d	65		HCC	-/-	-/-	610/1200
		5433M/4 Y	69		T	-/-	+/-	55/55
		6825H/7 y	72			-/-	+/-	ND/55
								
M/C	1205-15	5399M/7 y	35		HCC	-/-	-/+	32/28

ND = Not done, HCC = Hepatocellular carcinoma, T = tumor tissue only.

### 8 bp delections represent 8 % of the total samples

Four samples of the 50 samples (1 from group B and 3 from group C) presented 8 bp deletions at positions 389 to 397 nt of the HBx gene; the core promoter region, corresponding to 1763–1770 nt of the complete genome. Fig 2 shows the sequence and the band patterns of samples; 6541 (group C), 6290 (group C), 6516 (group C) and 467 (group B). To confirm that these deletions were not a PCR artifact, the samples were further digested by SspI. Of the four samples presenting the deletion only 2 were corroborated by SspI, both samples (467 and 6516) were re-amplified from the PCR1 product.

### Mutations observed in HBV acute infected patients that recovered versus chronic carriers

The percentage of the most frequent polymorphism found in the study as well as the consensus sequences of each of the population selected for statistical analysis are shown in Table 4. Group A presented more amino acid or nucleotide variability than the other groups, however, in acute phase samples from group B, 50% of these had common mutations at position 12 (T12A).

**Table 4:** Major sequence polymorphisms found in the groups studied.

Amino acid-Position-mutation	Frequency (%)	Consensus sequences
		
L5M	28	Group A: 17 recovered patients
Q8K	22	
T12A	36	
S29P	38	
S31P	30	
S33P	30	
V37I	33	
		
P40S	30	Group B: 7 acute-chronic patients MAARLCCQLDP-RDVLCLRPVGAESRGRSLSGSLGAVPPPSPSAVPADDGSHLSLRGLPV CSFSSAGPCALRFTSARRMETTVNAPRSLPTVLHKRTLGLSGRSMTWIEDYIKDCVFKDW EELGEEIRLKVFVLGGCRHKLVCSPAPCNFFTSA*
D48N	25	
R87W	30	
R103W	33	
		
T106P	25	Group B: 32 chronic patients MAARLCCQLDPTRDVLCLRPVGAESRGRSLSGSLGAVPPPSPSAVPADDGSHLSLRGLPV CSFSSAGPCALRFTSARRMETTVNAPRSLPTVLHKRTLGLSGRSMTWIEDYIKDCVFKDW EELGEEIRL- - FVLGGCRHKLVCSPAPCNFFTSA*
D110E	33	
K130M	24	
V131I	27	
		
Deleted nt	8	Consensus Deletion group 8 bp MAARLCCQLDPTRDVLCLRPVGAESRGRSLSGSLGAVPPPSPSAVPADDGSHLSLRGLPV CSFSSAGPCALRFTSARRMETTVNAPRSLPTVLHKRTLGLSGRSMTWIEDYIKDCVFKDW EELGEEIRLNIRRL*
390-397		
end codon		
135 aa		

Hyphens in the consensus sequence represent the amino acid polymorphism associated with the left column. The predicted consensus amino acid sequence was obtained with Bioedit Software from the nucleotide sequence of the sample study.

### Samples Genotype

Of the total 50 samples sequenced ; 48 belonged to genotype F, one sample belonged to genotype D subtype adw, and the other to subtype ay, which was classified as genotype E by a web-based genotyping tool and as D by phylogenetic tree analysis (data not shown).

## Discusion

T-A mutations were not found in any of 17 samples from HBV patients who had recovered; a similar result had been obtained in a study with self-limited acute hepatitis [20]. However, another study showed T-A mutations during the acute phase in one out of 11 from genotype A, none of the 5 patients from genotype B and 4 out of 27 from genotype C [21]. The significance of this finding needs to be further studied.

T-A mutations were found in 12 (41.3%) of 29 samples from chronic carriers. In one carrier the mutations were detected 29 days after onset, with the probability that this carrier could have been directly infected with HBV containing the T-A mutations. In the 23 acute phase samples, T-A mutations were not detected and therefore the possibility to have an initial infection with T-A in other populations appears to be low. However, Kobayashi *et al*, has shown in their study a higher prevalence of the T-A mutations in chronic patients during the acute phase than in acute self limited HBV infection in patients infected with genotypes C, A and B [21].

In chronic carriers, with a liver biopsy classified as moderate or severe, T-A mutations were present in 61.5% (8/13) and none in 4 biopsies classified as mild. However this result was not statistically significant based on the Fisher exact test, 1 tail, p = 0.05, probably due to the small sample size in the groups. Other studies have shown a better correlation between the presence of T-A mutations and patients with fulminant hepatitis, severe exacerbation [20] or liver cirrhosis [22] especially with genotypes A or C when compared with asymptomatic carriers [12-14]. In agreement with the literature T-A mutations seem to appear more frequently in genotypes C [23,24] and A [13] than D [25,26] or B [27].

In this study, V131I also occurred alone in 5 samples (17%) of the 29 chronic patients; this event has been commonly reported by others [6,14,25,27,28]; nevertheless M130K alone is very unusual. It has been described in 1 of 12 fulminant hepatitis patients [20] and in 1 genotype B strain [27]. In one of the paired samples from this study and in another from reference [6], the V131I mutation appears in time before the methionine change at position 130.

In a Korean study T-A mutations were found in 32% (13/41) of HBV carriers, and a triple mutation G1714A, C1718T, A1721G was found in 27% (11/41) patients [4]. In our study wild type (wt) HBV strain nucleotide were found in the 1714 and 1718 positions, but the mutation A1721G was found in genotype F samples and not in two samples with other genotypes. Again, T-A mutations are common in all genotypes while other mutations seem to be more related to specific genotypes.

No association could be established between the presence of T-A mutations and HBeAg status (Table 3), similar to other published data [4,24]. Of the four samples with the 8 bp deletion only (467 and 6516), two were re-amplified from the PCR1 product and corroborated by enzyme restriction digestion, which demonstrates that the deletion was not a PCR artifact. This 8 bp deletion in the T-A site has been reported previously [6,8,9,27,29] and it has been associated with a low viral load [7,8,29]. Different clones isolated from several patients showed a heterogeneous population of strains including T-A mutations, wt strains as well as the 8 bp deletion. This could be a possible reason why we observed different results in amplified samples of the initial PCR products with an 8 deletion than in the reanalyzed two samples where the deletion was not detected.

An interesting fact is that these deletions alter the X open reading frame, changing K130N and introducing an isoleucine in the 131 site and a stop codon in the position135.

The polymorphic differences observed between the sequence of acute HBV recovered patients and chronic carriers are related to the genetic diversity of strain more than the study group classification (A,B,C). All sequences isolated in this study belong to genotype F with the exception of 2. Using blast searches sequences from genotype F can be divided in AY090455 – 1889 NIC sequences similar to those which are related to South American sequences and the AY090456- 1980HCR sequences similar to those which are related to Central America sequences. The polymorphism observed in the nucleotides as well as the amino acids in these groups may be due to a variability present in the group related to the South American sequences.

Many efforts have been made in order to clarify the role of viral variants in the pathogenesis of HBV infection; and still there is no final consensus. T-A mutations have been proposed as possible prognostic markers for liver disease progression [14] however, more studies are needed to elucidate the role of the T-A mutations and its relation to HBV diversity and disease outcome.

## Conclusion

According to our results, T-A mutations were frequently observed in HBV chronic carriers, but were not found in acute recovered patients.

T-A mutations are frequent in all genotypes while other mutations seem to be more related to specific genotypes.

T-A mutations may appear early during HBV infection although the possibility of initial infection cannot be excluded.

## Methods

### Study population

Samples were obtained from a study of HBV in San Ramón and Palmares, Costa Rica areas outside of the capital city, San José, between 1972–1985 [19]. Based on serological markers and history of clinical onset, three groups were established: Group A, included 18 samples from acute cases who recovered from the infection; they presented initially as HBsAg positive, anti-IgM HBc positive and had elevated ALT levels. A patient was catalogued as a chronic carrier if HBsAg was present more than 6 months after the onset of disease. Group B, included 14 paired samples from chronic patients with known onset; with at least 3 years difference between the samples. Group C included 45 chronic patients with unknown date of onset. Twenty-nine patients had liver biopsy results, 4 from group B and 25 from group C.

The samples from all groups were negative by anti HAV IgM or anti- HCV [31] and were kept frozen.

This project was approved by the Ethical Committee of the Universidad of Costa Rica.

### Biopsy classification Pathology

The inflammatory activity of Knodell in Chronic Persistent Hepatitis (CPH) between 1 and 2 points, is represented by a uniform and diffuse cobblestone arrangement of swollen hepatocytes, with compressed sinusoids; some of which show Hadziyannis cells containing abundant HBsAg.

Lobular Chronic Hepatitis (LCH) is between 2 and 6 points with an intact lobular architecture, perivenular cell swelling, focal hepatocytolysis and a variable degree of inflammatory activity [32]. Further, these lesions are characterized by focal necrosis, abnormal hepatocytes and scattered passive fibrous interportal bridges.

In this study the Knodell Index (KI) was used as follows: ≤ 2 points was considered mild liver lesion, 3 and 4 moderate and > 4 as severe liver damage.

### PCR Methods

Primers were chosen from conserved regions of the following HBV genotypes sequence obtained from GenBank. Genotype A subtype adw2 (AF297625) and (AF373066), genotype B (AF121243), genotype C subtype adr (AB033550), subtype adw (AB033557), genotype D subtype ayw (AF280817), genotype E (AB032431), genotype F (AB036919), genotype G (AB064310) and (AF160501).

Outer primers selected were: sense (1182–1200) 5'GTTTGCTGACGCAACCCCC3' and the antisense 5'CAATGTCCATGCCCCAAAGC3' (1891–1910). The expected amplified product size was 728 bp. Inner primers: sense 5'GATCCATACTGCGGAACTCC3' (1263–1282) and antisense 5'AGCTTGGAGGCTTGAACAGT3' (1859–1878).

Genomic DNA was extracted from 200 μl of serum using the QIAamp DNA mini Kits (Qiagen^® ^U.S.A.) according to manufacturer's instructions.

Nested PCR was performed using a thermocycler (Perkin-Elmer).

For the first PCR, 10 μl of the extracted product were added to a total of 50 μl of reaction volume containing 2.5 units of Taq (Promega^® ^5 units/μl), 3.5 mM of MgCl_2_, 0.092 nmoles/μl of primers final concentration, 0.4 mmolar/μl of each dNTP. This amplification was performed at 94°C for 3 min followed by 40 cycles at 94°C for 1 min, 50°C for 1 min and 72°C for 1 min, with a final extension of 4 min to 72°C.

For the nested PCR, 5 μl of product from the first PCR were added to 50 μl of reaction, with a final concentration of MgCl_2_, 2.5 mM and 0.080 nM of primers. Cycling conditions for the second round were 94°C for 3 min, 40 cycles to 94°C for 0.40 min, 55°C for 0.40 min and 72°C for 1.30 min. The final extension was 72°C for 4 min.

Nested products with a size of 616 bp were corroborated by 2% agarose gel electrophoresis stained with ethidium bromide.

Dilutions of 1:10 of a commercial CPG^® ^DNA plasmid with 10^5^copies/μl of the total HBV genome were prepared and used as control as well as to determine the limit detection (sensitivity) of the PCR system.

### Sequencing conditions

Nested PCR product (616 bp) was run on 1% agarose gels and the expected band was cut and purified by a Qiagen column system following manufacturer's instructions.

An Open Gene™ sequencer system (Visible Genetics) was used. For sequencing the following primers were labeled with cy 5.0 and cy 5.5 dyes: Sense 5' 5cy55 GTTTYGCTCGCAGCMGGTC3' y = c/t, m = c/a (1292–1310) and antisense 5'-5cy5 CTTGAACGATRGGACATGAAC3' R = a/g (1848–1868).

Primers were diluted to a concentration of 3 pM in TE buffer. All reagents were used according to manufacturer's instructions. The first denaturation step was at 94°C for 2:30 min followed by 35 cycles of 0:30 min at 94°C, 0:30 min at 50°C, 1 min at 70°C and a final extension step at 72°C for 7 min. Finally, 1.5 μl of each sample was run in a polyacrylamide gel at 1500 volts for 90 min.

A consensus sequence of the genotype F strain (NCBI AB036919, AB036905, X75658) was used as our wild type sequence.

### Genotype sequencing

The HBx gene sequences were compared with homologue sequences obtained from the GeneBank data base using the BLAST program [33]. The genotype was determined using a web- based genotyping tool for viral sequences [34]. The subtype of some of the samples was determined previously by specific antibodies available in our laboratory.

### Restriction Enzyme digestion

In order to corroborate an 8 bp deletion observed in some sequences, a restriction enzyme SspI was used (New England, BioLabs _INC,_). As a positive control a sample from HIV having the same recognition site was used and a HBx sample with the wild type sequence was employed as a negative control. Ten μl of each purified product from the nested PCR were dispensed into two different vials of 200 μl. In one vial 1 μl of SspI enzyme (5000 units/ml), 2 μl of enzyme buffer (New England, BioLabs _INC,_) and 7 μl of water were added; while in the other vial the enzyme was omitted. All samples were heated at 37°C for 90 minutes and run in a 3% agarose gel. Results were visualized with ethidium bromide.

### Statistical analysis

The Fisher's exact test was used to evaluate the relationship between two discrete and dichotomy variables. The t test, for independent samples, was used to analyze continuous variables when it was necessary. A new dichotomy variable for hepatic damage was built into biopsy results and using data from the Costa Rican National Tumor Registry (NTR); by division into "mild damage" and "moderate/severe damage". The relative risk (RR) was calculated with a 95% confidence interval. All analyzes were done with the JMP 4 software version 4.0.4 A BUSINESS UNIT OF SAS Copyright ^© ^1989 – 2001 SAS Institute Inc. (all rights reserved) and Epiinfo software CDC.

## Competing interests

The author(s) declare that they have no competing interests.

## Authors' contributions

BL, FA, KV experimental design planning research

BL, MV laboratory: molecular and pathology work, respectively

BL, FA statistical analysis

BL, KV editing

LH, LT, RBL contributed to manuscript content and editing of drafts

## References

[B1] Beasley RP, Hwang LY, Lin CC, Chien CS (1981). Hepatocelullar carcinoma and hepatitis B virus: a prospective study of 22707 men in Taiwan. Lancet.

[B2] Bergametti F, Sitterlin D, Transy C (2002). Turnover of Hepatitis B Virus X protein is regulated by a Damaged DNA-Binding Complex. J Virol.

[B3] Lee H, Yun Y (1998). HBx protein of Hepatitis B virus activates Jak1-STAT signaling. J Biol Chem.

[B4] Chun YK, Kim JY, Woo HJ, Oh SM, Kang I, Ha J, Kim SS (2000). No significant correlation exist between core promoter mutations, viral replication and liver damage in chronic hepatitis B infection. Hepatology.

[B5] Buckwold VE, Xu Z, Chen M, Yen TS, Ou JH (1996). Effects of a naturally occurring mutation in the hepatitis B virus basal core promoter on precore gene expression and viral replication. J Virol.

[B6] Horikita M, Itoh S, Yamamoto K, Shibayama T, Tsuda F, Okamoto H (1994). Differences in the entire nucleotide sequence between hepatitis B virus genomes from carriers positive for antibody to hepatitis B e antigen with and without active disease. J Med Virol.

[B7] Sallam TA, Tong CY (2002). Two distinct types of hepatitis B virus core promoter variants in Yemeni blood donors. J Med Virol.

[B8] Schlager F, Schaefer S, Metzler M, Gratzki N, Lampert F, Gerlich WH, Repp R (2000). Quantitative DNA fragment analysis for detecting low amounts of hepatitis B virus deletion mutants in highly viremic carriers. Hepatology.

[B9] Preikschat P, Gunther S, Reinhold S, Will H, Budde K, Neumayer H, Kruger DH, Meisel H (2002). Complex HBV populations with mutations in core promoter, C gene and Pre S region are associated with development of cirrhosis in long-term renal transplant recipients. Hepatology.

[B10] Sirma H, Giannini C, Poussin K, Paterlini P, Kremsdorf D, Brechot C (1999). Hepatitis B Virus X mutants, present inhepatocellular carcinoma tissue abrogate both the antiproliferative and transactivation effects of HBx. Oncogene.

[B11] Hsia CC, Yuwen H, Tabor E (1996). Hot spot mutations in hepatitis B virus X gene in hepatocelullar carcinoma. Lancet.

[B12] Fang ZL, Ling R, Wang SS, Nong J, Huang CS, Harrison TJ (1998). HBV core promoter mutations prevail in patients with hepatocellular carcinoma in Guangxi, China. J Med Virol.

[B13] Batista M, Kramvis A, Kew M (1999). High prevalence of 1672T 1764G mutations in the basic core promoter of Hepatitis B virus isolated from black Africans with hepatocellular carcinoma compared with asymptomatic carriers. Hepatology.

[B14] Lindh M, Hannoun C, Dhillon AP, Norkrans G, Horal P (1999). Core Promoter Mutations and Genotypes in Relation to Viral Replication and Liver Damage in East Asian Hepatitis B Virus Carriers. J Inf Dis.

[B15] Norder H, Hammas B, Lee SD, Courouce AM, Mushahwar IK, Magnius L (1993). Genetic relatedness of hepatitis B viral strains of diverse geographical origin and natural variations in the primary structure of the surface antigen. J Gen Virol.

[B16] Norder H, Courouce AM, Magnius LO (1994). Complete genomes, phylogenetic relatedness, and structural proteins of six strains of the hepatitis B virus, four of which represent two new genotypes. Virology.

[B17] Arauz-Ruiz P, Norder H, Robertson BH, Magnius L (2002). Genotype H: a new American genotype of hepatitis B virus revealed in Central America. J Gen Virol.

[B18] Arauz-Ruiz P, Norder H, Visoná K, Magnius L (1997). Genotype F prevails in HBV infected patients of Hispanic origin in Central America and may carry the precore stop mutant. J Med Virol.

[B19] Visoná K, Eduarte C, Zamora E, Salazar L (1989). Estudio epidemiológico de las hepatitis virales en San Ramón y Palmares de 1972–1985. Acta Médica Costarricense.

[B20] Honda A, Yokusaka O, Suzuki K, Saisho H (2000). Detection of mutations in hepatitis B virus enhancer 2/core promoter and x protein regions in patients with fatal hepatitis B virus infection. J Med Virol.

[B21] Kobayashi M, Arase Y, Ikeda K, Tsubota A, Suzuki Y, Saitoh S, Kobayashi M, Suzuki F, Akutas N, Hosaka T, Someya T, Matsuda M, Sato J, Miyakawa Y, Kumada H (2004). Wild type precore and core promoter sequences in patients with acute self limited or chronic Hepatitis B. Scand Journal Gastroenterol.

[B22] Cho SW, Shin YJ, Hahm KB, Jin JH, Kim YS, Kim HJ (1999). Analysis of the precore and core promoter DNA Sequence in liver tissues from patients with Hepatocellular carcinoma. J Korean Med.

[B23] Fang ZL, Yang J, Ge X, Zhuang H, Gong J, Li R, Ling R, Harrison TJ (2002). Core promoter mutations (A_1762 _T and G_1764 _A) and viral genotype in chronic hepatitis B and hepatocellular carcinoma in Guangxi, China. J Med Virol.

[B24] Ni YH, Chang MH, Hsu HY, Tsuei DJ (2004). Longitudinal study on mutation profilies of core promoter and precore regions of the hepatitis B virus genome in children. Ped Res.

[B25] Vernard V, Corsaro D, Kajzer C, Bronowicki J, Faou A (2000). Hepatitis B virus X gene variability in French-born patients with chronic hepatitis and hepatocellular carcinoma. J Med Virol.

[B26] Hannoun C, Horal P, Lindh M (2000). Long -term mutation rates in the hepatitis B virus genome. J Gen Virol.

[B27] Bläckberg J, Kidd-Ljunggren K (2003). Mutations within the hepatitis B virus genome among hepatitis B patients with Hepatocellular carcinoma. J Med Virol.

[B28] Gandhe SS, Chadha MS, Walimbe AW, Arankalle VA (2003). Hepatitis B virus: prevalence of precore/core promoter mutants in different clinical categories of indian patients. Viral Hepatitis.

[B29] Li KS, Yamashiro T, Sumie A, Terao H, Mifune K, Nishizono A (2001). Hepatitis B virus harboring nucleotide deletions in the core promoter region and genotype B correlate with low viral replication activity in anti-HBe positive carriers. J Clin Virol.

[B30] Kuang SY, Jackson PE, Wang JB, Lu PX, Munoz A, Qian GS, Kensler TW, Groopman JD (2004). Specific mutations of hepatitis B virus in plasma predict liver cancer development. Proc Natl Acad Sci U S A.

[B31] Palacios A, Taylor L, Haue L, Luftig RB, Visona KA (1999). Development of low cost peptide-based anti-Hepatitis C virus screening and confirmatory assays: Comparison with commercially available tests. J Med Virol.

[B32] Peters RL, Okuda K, Peters RL (1976). Hepatocellular carcinoma.

[B33] Altschul SF, Gish W, Miller W, Myers E, Lipman D (1990). Basic local alignment search tool. J Mol Biol.

[B34] Rozanov M, Plikat U, Chappey C, Kochergin A, Tatusova TA (2004). Web-based genotyping resource for viral sequences. Nucleic Acids Research.

